# Centrosome and retroviruses: The dangerous liaisons

**DOI:** 10.1186/1742-4690-4-27

**Published:** 2007-04-14

**Authors:** Philippe V Afonso, Alessia Zamborlini, Ali Saïb, Renaud Mahieux

**Affiliations:** 1 Unité d'Epidémiologie et Physiopathologie des Virus Oncogènes, CNRS URA 3015, Département de Virologie, Institut Pasteur, 28 rue du Dr Roux, 75015 Paris, France; 2 CNRS UMR7151, Hôpital Saint-Louis, 1 Avenue Claude Vellefaux, 75475 Paris Cedex 10, France

## Abstract

Centrosomes are the major microtubule organizing structures in vertebrate cells. They localize in close proximity to the nucleus for the duration of interphase and play major roles in numerous cell functions. Consequently, any deficiency in centrosome function or number may lead to genetic instability. Several viruses including retroviruses such as, Foamy Virus, HIV-1, JSRV, M-PMV and HTLV-1 have been shown to hamper centrosome functions for their own profit, but the outcomes are very different. Foamy viruses, HIV-1, JSRV, M-PMV and HTLV-1 use the cellular machinery to traffic towards the centrosome during early and/or late stages of the infection. In addition HIV-1 Vpr protein alters the cell-cycle regulation by hijacking centrosome functions. Enthrallingly, HTLV-1 Tax expression also targets the functions of the centrosome, and this event is correlated with centrosome amplification, aneuploidy and transformation.

## Background

### I. Centrosome functions

#### Centrosome and cell organization

Centrosomes were first described at the end of the 19th century by Theodor Boveri who had also the intuition of their central role in cell life [1].

Centrosomes are animal-specific non-membranous organelles that localize in close proximity to the cell nucleus for the duration of interphase. Their structure is highly conserved among higher eukaryotes. It usually consists of a pair of centrioles joined by fibers connecting their proximal ends which are embedded into a protein-dense matrix called the pericentriolar material (PCM) [2,3]. The PCM is an ordered lattice that anchors a large number of microtubule (MT)-associated proteins, many of which bear putative coiled-coil domains, a tertiary structure known to facilitate protein-protein interactions [4]. Centrioles are cylindrical corps formed by a radial array of nine MT-triplets, which are structurally similar to basal bodies of eukaryotic cilia and flagella [5,6]. Centrioles play a role in the organization of the microtubular cytoskeleton, but they do not make direct contact with the MTs which nucleate from the γ-tubulin ring complexes (γ-TuRC) located within the PCM.

In animal cells, centrosomes represent the major microtubule-organizing structures (MTOC). The MTOC is responsible to direct the assembly and the orientation of MTs and to control MT-dependent processes such as trafficking of cytoplasmic vesicles and orientation of cellular organelles. At the onset of mitosis, centrosomes become the core structures of spindle poles and direct the formation of mitotic spindles. Upon cytokinesis, each daughter cell receives only one centriole, which duplicates once per cell cycle.

#### Centrosome duplication and mitotic progression

**Figure 1:**
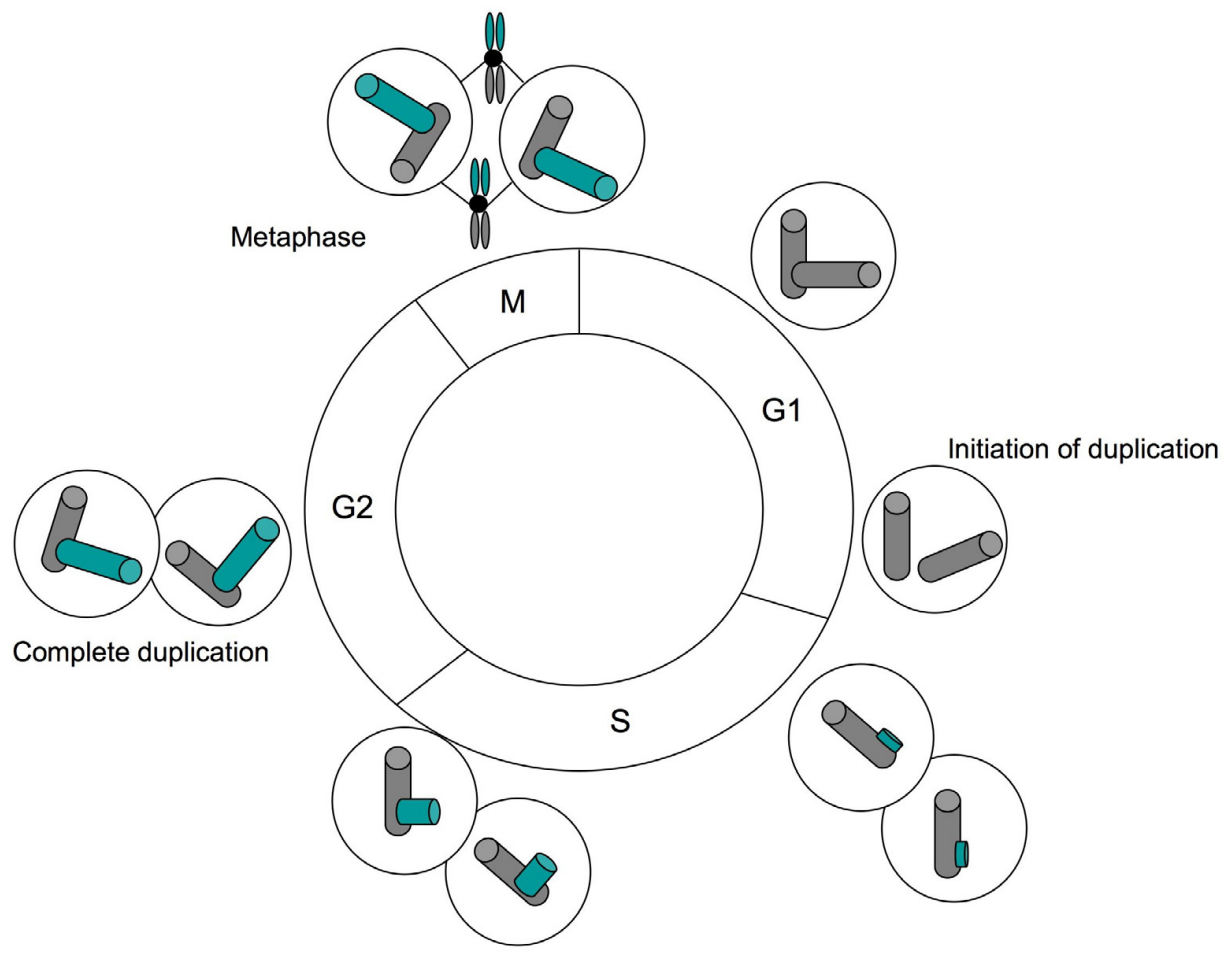
**The centrosome duplication cycle**. Adapted from [3, 14, 107]. Centrosome duplication starts at the G1/S transition with the separation of the paired centrioles. Procentrioles form near the proximal ends of each pre-existing centriole. Procentriole formation is completed during S phase. During mitosis the two centrosomes are present and form the mitotic spindle poles. Each cell inherits one centrosome after the completion of cytokinesis.

The number of centrosomes within a cell is strictly controlled [5] (Figure 1). In G1 phase, cells have a single centrosome consisting of two centrioles joined by cohesion fibers. At the G1/S transition, new centrioles grow orthogonally from each of the two pre-existing ones. They will elongate until G2, maintaining the strictly perpendicular configuration [5-7]. In early mitosis, the cohesion between the two pairs of centrioles is broken and each of them participates in the formation of the mitotic spindle pole.

Centrosome duplication is tightly regulated and limited at once *per *cell cycle through a mechanism that prevents re-duplication. The complexes between Cyclin-dependent kinase 2 (Cdk2) and either Cyclin E or Cyclin A (Cdk2/CycE/CycA) and their substrate, nucleophosmin, have been proposed to act as licensing factors for centrosome duplication [8,9]. However, this hypothesis has been recently challenged by Tsou and Stearns [5] who proposed that re-duplication is inhibited by a centrosome-intrinsic block [10]. They suggested that the engaged/orthogonal conformation of the centrioles functions as a block for re-duplication. The cellular factor which promotes centriole-disengagement at the end of mitosis would therefore constitute the licensing factor [5].

Additionally several mitotic kinases of the Aurora, Polo and Nek families, which localize at least temporally to mitotic structures, have been shown to participate in the control of the centrosome cycle and mitotic spindle formation [11].

The tight control of centrosome number and duplication is essential for the stability of the genome. Consequently, any impairment in the regulation of centrosome number might lead to the assembly of multipolar spindles [12], which in turn might increase the frequency of aberrant mitosis and chromosome-segregation errors [3].

Recent studies demonstrated that MTOCs play a key role in cellular processes other than nucleation and organization of the MT network. Indeed, the centrosome provides a subcellular site where high local concentration of regulatory molecules in the proximity of their substrates is likely to increase the probability of specific interactions. Many regulatory molecules localize, at least temporally, at the centrosome. Thus, it has been suggested that the centrosome might act as a scaffold platform where integration of numerous cellular signaling pathways occurs, including control of cell cycle progression and completion of cytokenesis [13,14].

As an example, following their injection into G2-arrested oocytes, centrosomes induce cell progression into mitosis [15]. Likewise, in a *Xenopus *egg model, centrosomes can induce the activation of the mitosis promoting factor (MPF or Cyclin dependent kinase 1 (Cdk1) and Cyclin B complex), which is a major event in the initiation of mitosis [16]. In mammalian cells, activation of MPF takes place at the centrosome during prophase and before any MPF-dependent H3-phosphorylation is detected in the nucleus [17]. MPF activity is controlled by cyclin phosphorylation through the antagonistic actions of Cdc25 and Wee1, which are also regulated by phosphorylation [18,19]. Finally, several positive and negative mitotic regulators which have distinct localization along the cell cycle, are also found at the centrosome during early mitosis (reviewed in [14]).

#### Centrosome amplification

Centrosome amplification (more than three centrioles in a cell during the G1 phase) can result from different mechanisms: (a) duplication of centrosome more than once during a cell cycle, (b) failure of cell cytokenesis, (c) uncontrolled splitting of a centriole pair and (d) formation of acentriolar MTOCs [3]. Centrosome amplification is often associated with genomic instability and therefore aneuploidy. Aneuploidy (i.e. the acquisition or loss of one or more chromosome from a diploid genome) is a very common feature of tumor cells [20-23]. After Boveri's hypothesis (the "aneuploidy hypothesis"), it has been admitted that cancer cells become aneuploid as a consequence of anomalous mitotic divisions. These defects were thought to result from centrosome amplification and transformation, and aneuploidy appeared likely to promote tumorigenesis, at least at low frequency. However, after the discovery of tumor suppressors and oncogenes, this assumption became debated [24-26].

A number of facts still support this hypothesis: first, aneuploidy frequently occurs before transformation. This is the case in a number of pre-cancerous lesions (cervix, colon, oesophagus etc.) [27-29]. Next, aneuploidy results in the de-regulation (up- or -down) of the expression of a number of genes. Third, transformation linked to aneuploidy requires several generations, which is coherent with the known incidence of cancers with age (for a review see [30]). Finally *in silico *modeling confirmed that the "aneuploidy theory" could explain how lymphocytes become transformed [31].

In the end, it was still technically challenging to test whether aneuploidy causes cancer or not until recently, since causing aneuploidy usually results in other cellular defects. Interestingly, in a very elegant study aimed at understanding whether aneuploidy drives tumorigenesis, contributes to tumor progression or is benign, Weaver and coll. demonstrated that aneuploidy acts both oncogenically and as a tumor suppressor [32]: Low levels of chromosomal instability promote tumor initiation while higher levels are protective [32].

### II. Retroviruses infection and centrosomal functions alteration

#### Hijacking centrosomal functions during entry and assembly: the FV, HIV-1 HTLV, M-MPV cases

**Figure 2:**
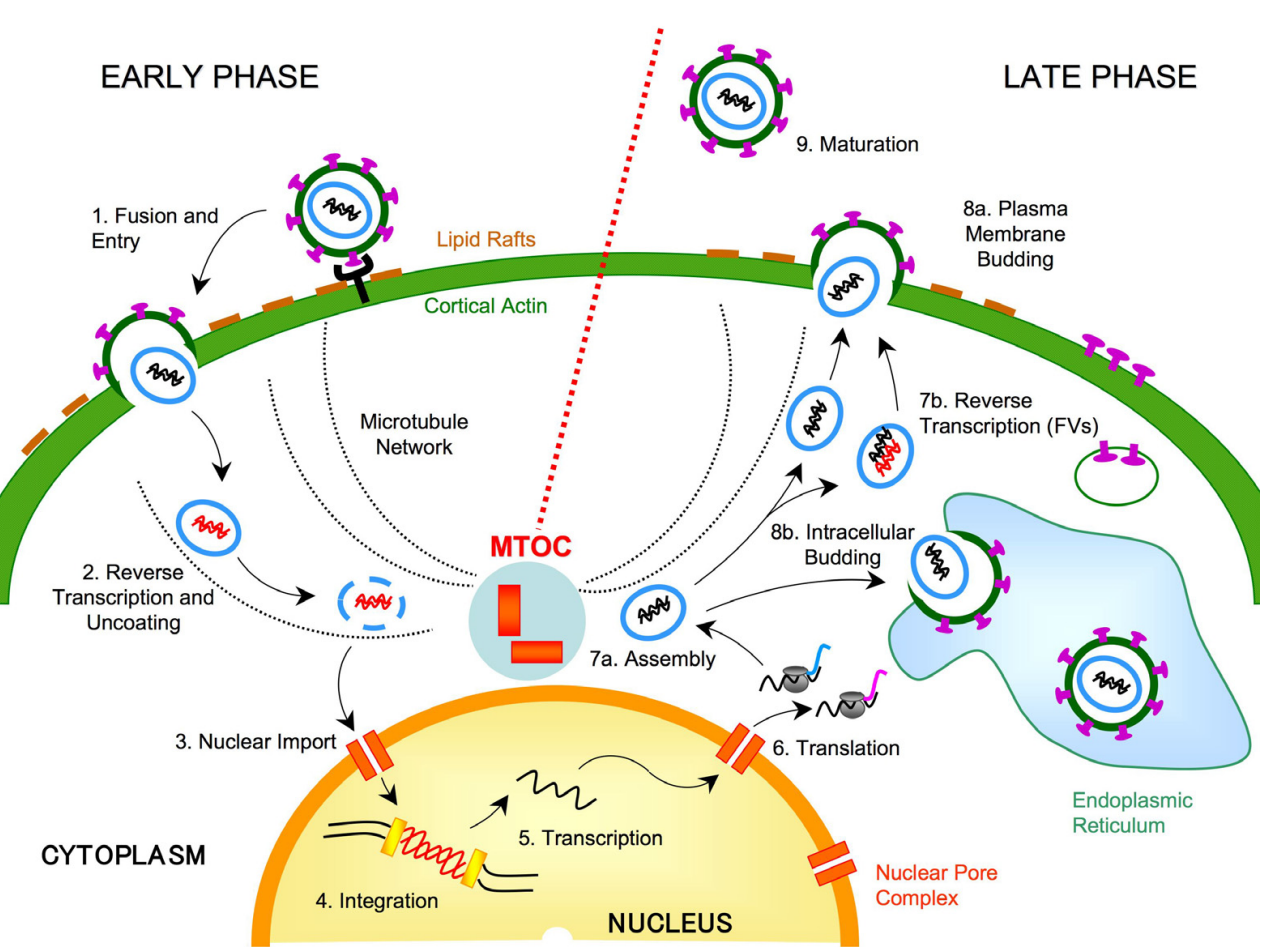
**Retroviruses target the MTOC during the early and/or the late phase of the viral replication cycle**. Retroviruses enter into the host cell mainly by receptor-mediated fusion of the viral envelope with the plasma membrane (1). After crossing the actin cortex, the viral core is released into the cytoplasm where it undergoes a process of uncoating during which the viral genomic RNA (black) is reverse transcribed into a double-strand linear DNA copy (red) (2). Incoming viral cores *en route *to the nucleus reach the MTOC by using the molecular motor complexes to traffic along the MTs (3). In the nucleus the viral DNA genome is stably integrated into the host cell chromosome (4). The integrated viral DNA or provirus is the template for the synthesis of the viral mRNAs (5) which are transported in the cytoplasm and translated to produce the viral Gag polyproteins and the viral envelope glycoproteins(6). Newly synthesized Gag proteins and the viral genomic RNA converge to the MTOC where encapsidation and assembly initiate (7a). At this late stage, FVs are characterized by a second reverse transcription event (7b). By trafficking along the MT network assembling viral particles reach the plasma or endosomal membranes where budding occurs (8a and b). Finally, for most retroviruses, a process known as maturation is necessary for the generation of infectious viruses able to begin a new round of infection (9).

The movement of viruses through the cytoplasm – a highly viscous milieu that consistently limits directional movement by free diffusion – relies on the cellular active transport system [33]. Viruses cross twice the cytoplasm during the course of the infection, after entry to get to the site of replication and to reach the sub-cellular location where assembly of new progeny virions and budding occurs (reviewed in [34-36]) (Figure 2). Since MTs originate as radial array from the MTOC in most cells, with their minus ends anchored to the PCM and the plus ends extending towards the cell periphery, it is not surprising that several viruses, among which retroviruses, have been found to concentrate near the centrosome in a MT-dependent manner on their way to and/or from the nucleus. To date, several potential direct interactions between viral components and MT-motors have been reported and it has been established that, to traffic into the cytoplasm, viruses have evolved two alternative strategies, either by hijacking cytoplasmic vesicles or by directly interacting with MT-associated molecular motors.

#### Entry

Foamy Virus (FV) and Human Immunodeficiency Virus type 1 (HIV-1) were reported to target the centrosome at an early phase of the viral life-cycle. Soon after entry in the host cell, both FV [37,38] and HIV-1 [39-41] incoming virions *en rout*e to the nucleus accumulate in the peri-centrosomal area, in a MT and dynein-dynactin dependent manner (Figure 2).

An N-terminal coiled-coil motif within FV Gag (aa 150–180), is responsible for the centrosomal localization of the viral particles at this stage [37]. In G0-arrested cells both FV Gag and the viral genome persist at this location for several weeks. Remarkably, FV life cycle resumes as soon as infected quiescent cells are stimulated to divide: integration and gene expression occur leading to the formation and the release of new progeny virions (Lehmann-Che submitted). Maintenance of viral capsids at the centrosome in quiescent cells could be a strategy that FVs have evolved to rapidly respond to stimuli received by the infected cell.

HTLV-1 requires cell-to-cell contact to spread efficiently. Interestingly, it has been observed that the MTOC of an HTLV-1-infected cell, which is involved in the formation of a two-cell conjugate, relocates in proximity of the adhesion site. In addition HTLV-1 genome and the viral proteins Gag and Env concentrate in the same area. Therefore, it has been suggested that polarization of the MTOC results in the orientation of many MT-plus ends towards the cell-cell junction, thus allowing the recruitment of viral Gag-containing complexes in proximity of the virological synapse where viral transmission ultimately takes place [42].

#### Assembly

FV capsids form in the cytoplasm in the vicinity of the MTOC (Figure 2). Assembly depends on the integrity of the cytoplasmic targeting-retention signal (CTRS) sequence within Gag, which is responsible for the peri-centrosomal targeting of the polyprotein [43]. The CTRS is an 18-amino acid long motif found within the MA domain of Gag, which functions as a dominant signal that directs intracytoplasmic capsid assembly of B/D-type retroviruses (such as FV and Mason-Pfizer Monkey Virus (M-PMV)) despite the presence of the bipartite membrane-targeting signal at the N-terminus of Gag [44].

Early work by Hunter and coll. showed that M-PMV, the prototypic D-type retrovirus, assembles at a perinuclear location [45], which has recently been identified as the MTOC [46]. Specific targeting of M-PMV Gag polyproteins to the pericentriolar region is mediated by the CTRS, which interacts in a co-translational manner with the dynein-dynactin motor complexes [46]. Nascent Gag polyproteins still associate to the polysomes, accumulate near the centrosome where both partially and fully assembled spherical capsids are visualized by electron microscopy. Gag was suggested to interact with endocytosed M-PMV Env glycoproteins trafficking into pericentriolar recycling endosomes at this subcellular location, thus allowing efficient migration of the immature capsids towards the budding sites at the plasma membrane [47]. Of note, centrosomal targeting does not appear to be absolutely required for M-PMV assembly: Indeed, a point mutation (R55W) within the CTRS abolishes centrosomal accumulation of M-PMV Gag. Yet, Gag mutant still assembles at the plasma membrane following a type C morphogenesis pattern [48].

Similarly, the Gag polyprotein of Jaagsiekte Sheep Retrovirus (JSRV), another β-retrovirus localizes near the MTOC of infected cells and its centrosomal targeting is a prerequisite for the subsequent transport of JSRV virions to the plasma membrane but not for assembly [49]. Indeed co-expression of Gag from JRSV and enJS56A1, a sheep endogenous retrovirus closely related to JSRV, results in the delocalization of JSRV Gag from the MTOC. Chimeric viral particles composed of both JSRV and enJS56A1 Gag polyproteins still assemble in the cytoplasm but they cannot reach the plasma membrane [49].

The centrosome has been proposed to be the subcellular site where HIV-1 Gag polyproteins are synthesized and bind to the viral genomic RNA, thus initiating encapsidation and viral particle assembly. Indeed siRNA-mediated depletion of heterogeneous nuclear ribonucleoprotein A2 (hnRNP A2), a cellular protein which expression levels regulate the nucleocytoplasmic trafficking of HIV-1 genomic RNA, almost results in the complete pericentriolar accumulation of the viral genomic RNA without affecting Gag-expression levels [50]. HIV-1 genomic RNA and newly synthesized Gag molecules colocalize near the centrosome in a manner that depends on the presence of the packaging signal region (ψ), a high-affinity binding site for Gag within the viral RNA genome. These observations suggest that ψ acts as a targeting signal which specifically directs initiation of HIV-1 encapsidation to this subcellular domain [51].

The centrosome has also been suggested to represent an advantageous site for virus assembly because of the high local concentration of chaperons [52,53] which have been shown to participate in M-PMV Gag folding and thus in viral capsid formation [54].

In addition, because of its role as a cellular MT-organizing center and its perinuclear localization, the centrosome represents an optimal site (1) through which incoming viruses can easily gain access to the nucleus and (2) through which newly synthesized viral components traffic to reach the viral assembly and budding sites (Figure 2). The precise mechanisms that viruses and viral components use to transit from the MTOC to the nucleus and then back are still under investigation, but it has been suggested that importins or the dynein-mediated trafficking might be involved in this process [14].

#### Cell cycle arrest, centrosome dysfunctionand apoptosis: the Vpr case

Early studies reported that HIV-1 14kDa Viral protein R (Vpr) was an oncogenic protein and suggested that it could be responsible for some AIDS-associated cancers [55]. At that time, several groups reported the presence of multiple centrosomes, and consequently aneuploidy as well as micronuclei in Vpr-expressing cells [56]. Even if it has now been clearly demonstrated that Vpr is not oncogenic *per se*, it still would be interesting to understand the molecular bases of such centrosomal dysfunctions.

**Figure 3:**
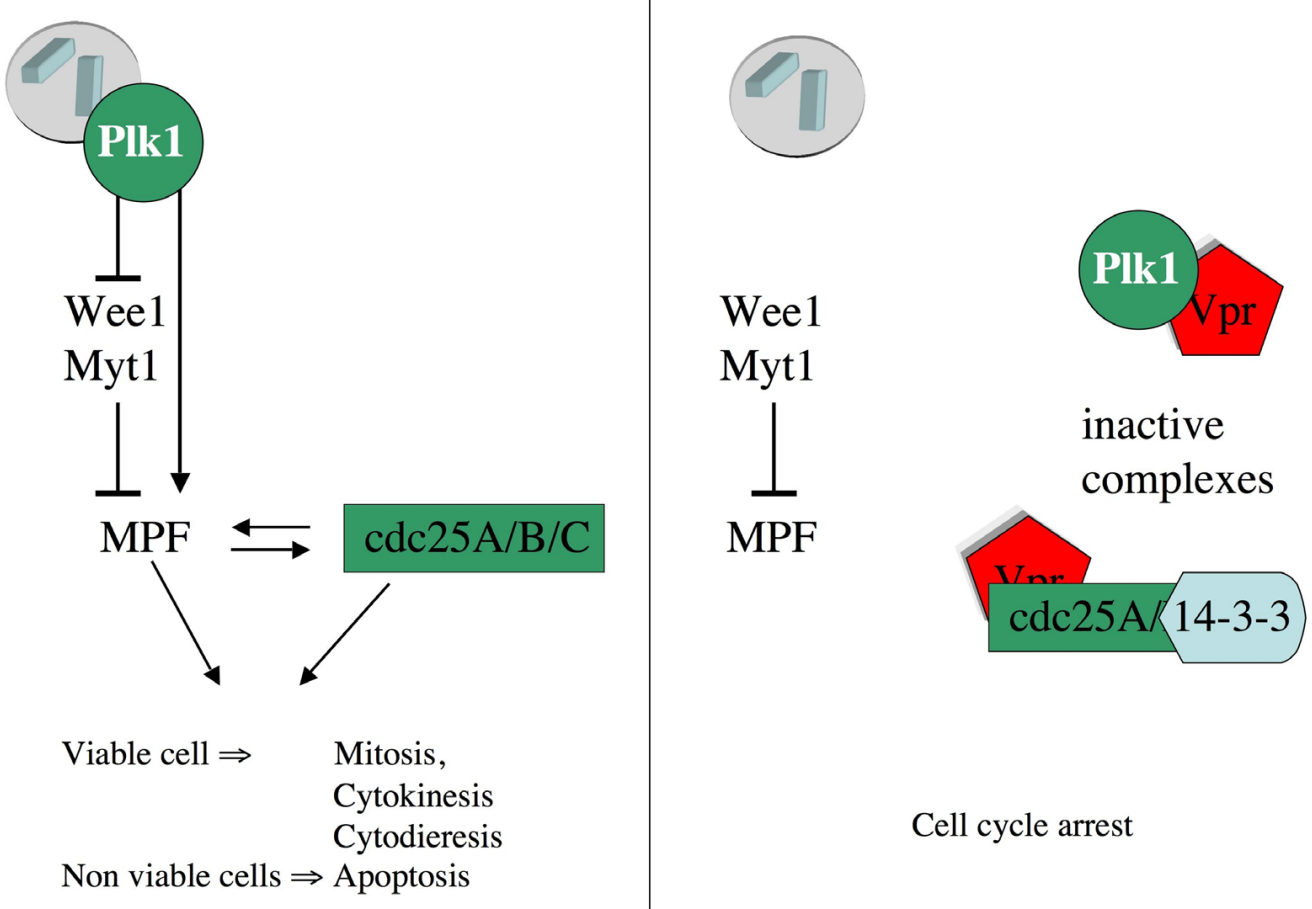
**Vpr induces a G2 cell cycle arrest and eventually apoptosis**. (A) In uninfected cells Polo like kinase-1 (Plk1) activates the Mitosis Promoting Factor (MPF) both directly and indirectly by inhibiting the Wee1 and the Mut1 kinases. Cdc25 proteins are also involved in MPF activation via a positive retroactive loop. In normal cells, MPF activation leads to mitosis; in non-viable cells, mitosis acts as a checkpoint and cells die by apoptosis. (B) In HIV-1 infected cells, Vpr expression induces the relocalization of Plk1 and form a ternary complex with 14-3-3 and Cdc25. MPF cannot be activated, and consequently cells arrest in G2 phase. Non-viable (polyploid) cells transiently accumulate. If the cell-cycle arrest is prolonged, cells die by necrosis.

Centrosomal amplification and subsequent aneuploidy might result from a direct alteration of centrosomal integrity by Vpr. It has been shown that Vpr induces the delocalization of the polo kinase Plk1/Plo1, a component of the centrosome, which is usually located on the spindle pole body (Figure 3). Depending on the cell line used for the experiment, Plk1/Plo1 localizes either in the cytoplasm forming dots or in the nucleus in Vpr-expressing cells [57]. Delocalization of Plo1 has been demonstrated to be independent of the G2 arrest. However, it is conceivable that Plo1 mislocalization, at least in yeast cells, might be a consequence of its direct interaction with Vpr, which localizes in the nucleus. Because Plo1 is a regulator of several aspects of cell division, including mitotic entry, mitotic spindle assembly, centrosome maturation, mitotic exit, and cytokinesis [58,59], its dissociation from the centrosome is likely to alter centrosomal functions (Figure 3). Nevertheless, it cannot be formally excluded that Vpr-associated polyploidy is due to the viral-induced alteration of the cell cycle, resulting in the uncoupling of the nuclear and the centrosomal cycles.

Indeed, HIV-1 infection impairs cell-cycle progression and infected cells accumulate in G2 *in vitro *[60]. Although it has been shown that several HIV-1 proteins independently block the cell cycle [61], Vpr is considered the major viral determinant responsible for such arrest. The G2 arrest might then allow the survival of abnormal cells, such as polyploid ones [60,61]. Noteworthy, the amount of Vpr present in a viral particle is sufficient to induce cell cycle arrest [62]. By mutational analysis, it has been demonstrated that the C-terminal domain of Vpr is responsible for the cell cycle arrest [63,64]. Moreover it has been established that Vpr phosphorylation is needed for this function [65]. Following Vpr-expression, the accumulation of G2-arrested cells correlates with the inactivation of the MPF, which normally occurs at the centrosome [57,66,67]. Vpr-mediated cell cycle arrest is accompanied by an hyperphosphorylation of Cdk1 [60,66,67]. The published results do not show any linear correlation between the amount of Vpr and the inhibition of MPF, which is consistent with the fact that Vpr does not bind directly to MPF components [68].

Rather, the viral-induced cell cycle dysfunctions seem to correlate mostly with an alteration of the functions of MPF regulators, such as Wee1 and Cdc25. This is supported by the finding that Vpr interacts with these proteins, at least in a yeast two-hybrid assay [68]. Cdc25 is a component of a family of phosphatases that activate MPF by antagonizing the effects of Wee1. It has been reported that Vpr alters Cdc25 activity either directly [69] or through the interaction with Cdc25-inhibitors such as 14-3-3σ and PPA2 [70,71] (Figure 3). 14-3-3 localizes at the centrosome during mitosis and inhibits the activity of Cdc25 by binding to the phosphorylated form of the protein [72]. Vpr-binding allows 14-3-3 to interact with the unphosphorylated form of Cdc25C. This trimeric complex delays the entry into mitosis [73,74] (Figure 3).

The prolonged cell cycle arrest induces apoptosis, which could be eventually responsible for the CD4+ depletion that is observed *in vivo *during AIDS progression [75].

#### HTLV-1: uncontrolled cell proliferation, transformation.... and aneuploidy

In contrast to HIV-1, which is responsible for the depletion of the CD4^+ ^cell population, HTLV-1, the etiological agent of Adult T cell leukemia/lymphoma (ATLL), causes lymphocyte transformation. Generally, lymphoma occurrence is linked to cell-cycle alterations. During the course of HTLV-1 infection, the alteration of the cell-cycle regulation is tightly linked to the expression of the viral protein Tax, which largely carries out the transforming capacity of HTLV-1. Tax can activate the NF-κB, the CREB/ATF and the SRF pathways. Both CREB/ATF and NF-κB pathways have been involved in the Tax-mediated immortalization/transformation (for a review see [76]).

The G1/S checkpoint regulation is tightly associated with the activation of the transcription factor E2F. In resting cells, E2F is inactive due to the formation of a complex with the Retinoblastoma protein (Rb). Once Rb is phosphorylated, either by the cyclinD-cdk4 complex at early G1, or by the cyclinD-cdk6 complex at late G1, it is degraded and E2F turns active. In Tax expressing cells, the G1/S transition is altered. A decade ago, Schmitt et al. demonstrated that the increased proliferation of Tax-expressing cells was correlated with an increased activity of both cdk4 and cdk6 [77]. Such activation has been since demonstrated to be a consequence of direct interaction of cdk4 and cdk6 with the amino-terminal domain of Tax [78,79]. Tax can also act directly on Rb. It binds directly to hypophosphorylated Rb and sends it to the proteasome where it is degraded [80].

Because it alters the MPF regulation, Tax can also disrupt the G2/M checkpoint. MPF regulation is dependent on Cdc25. The function of Cdc25 is modulated by Chk-proteins and the activity of both Chk1 and Chk2 is impaired in presence of Tax, *in vitro *and *in vivo *[81,82]. The action of Tax is a consequence of its ability to bind directly to these latter proteins. Consequently, in HTLV-1 infected cells, Cdc25 activity is not repressed and the progression into mitosis occurs earlier than scheduled [83].

Altogether, these cell-cycle alterations may facilitate the accumulation of errors during mitosis therefore inducing centrosomal alterations and aneuploidy.

This hypothesis is consistent with the reports of HTLV-1-leukemic cells carrying multi-lobulated nuclei (also known as "flower cells"). These cells possess an abnormal number of centrosomes and chromosomes [84-88]. The frequency of aneuploid cells is significantly increased in acute or classical ATLL patients, even if compared to chronic ATLL [86,89]. This is also accompanied by structural chomosomes abnormalities such as translocations and deletions. The cytogenetic abnormalities that are found in ATLL cells are not specific of these disease. They are however more frequent in the acute and lymphoma types than in chronic or smoldering types. They include various karyotypic abnormalities including translocations on chromosome 14 (14q32, 14q11), deletions of 6q but also numerical abnormalities such as trisomies 3,7 and 21 as well as monosomy of the × chromosome or loss of an Y chromosome [90].

As Tax is the HTLV-1 protein that alters the cell-cycle, its expression was suspected to be necessary and sufficient to induce aneuploidy and centrosome multiplication. Nitta et al. using JPX-9 cells that expresses Tax upon stimulation with cadmium [91] showed that Tax expression indeed allowed the accumulation of cells with an abnormal number of centrosomes [86]: 10% of Tax-expressing cells, displayed an abnormal number of centrosome, whereas only 5% of JPX-9 untreated cells showed centrosome amplification. Interestingly the authors also showed that the micronuclei formation was consecutive to the centrosome amplification and that up to 2% of Tax-expressing JPX9 cells exhibited micronuclei at 72 h of treatment.

**Figure 4:**
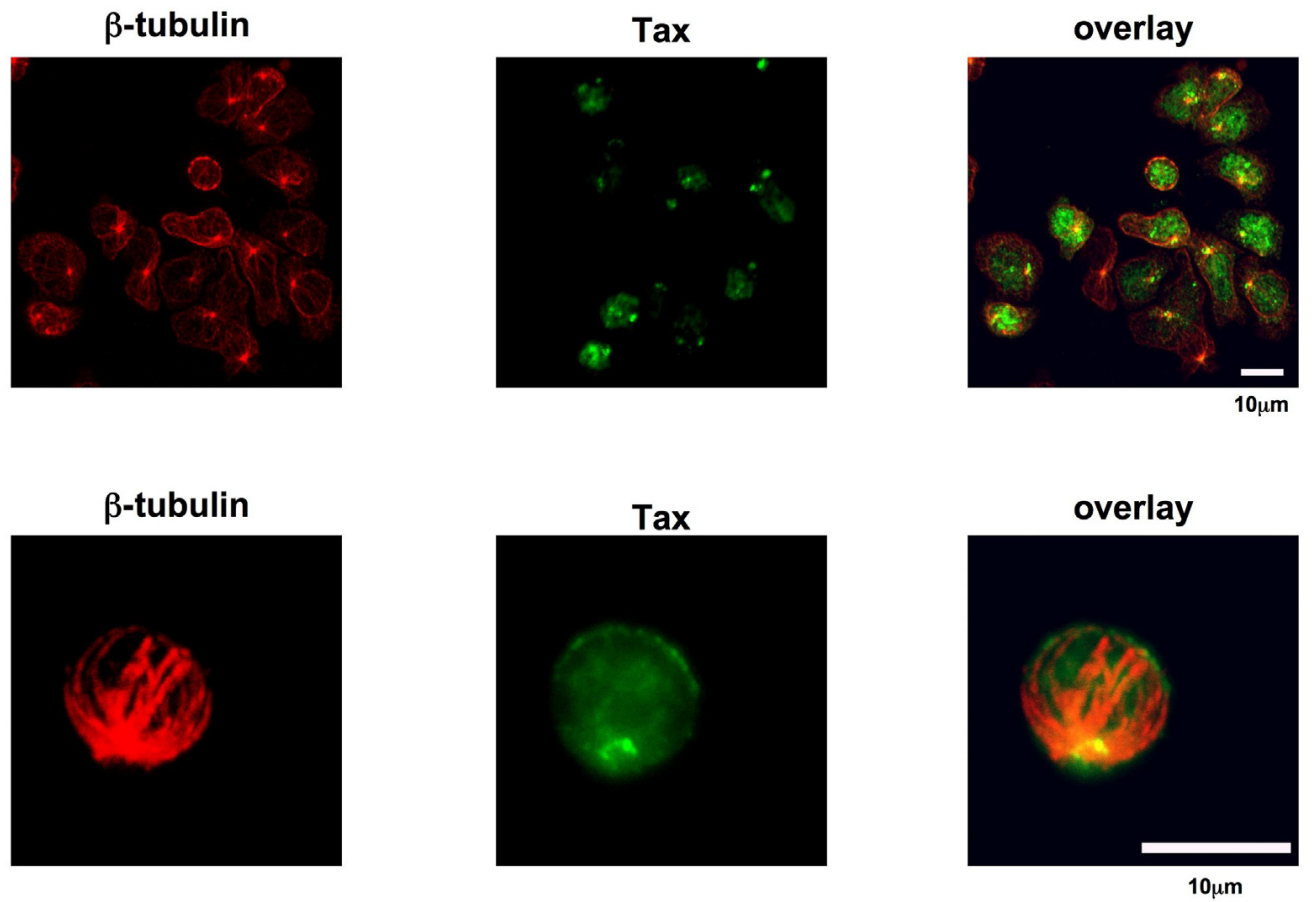
**In naturally infected T cells a substantial fraction of Tax co-localizes with the MTOC**. Images of CD4+ T cells naturally infected with HTLV-1 obtained from a TSP/HAM patient. Cells were stained with anti-Tax mAb Lt-4 (green) and monoclonal anti-β Tubulin-Cy3 antibody (red). The view represents a projection (XY axis) of adjacent confocal sections. Scale bar = 10 μM.

Several studies suggested that, besides an indirect role of Tax via cell-cycle regulation alterations, the viral protein could also be directly implicated in aneuploidy and centrosome amplification. If this was the case, then a fraction of Tax should be located close the centrosome in order to induce centrosome multiplication (Figure 4). In fact, in transfected mouse embryonic fibroblasts, Tax localizes at the centrosomes during the M phase [87]. Using a series of GFP-tagged Tax constructs, Peloponese and coll. also suggested that the Tax-dependent NF-kB activation is necessary to induce the presence of supernumerary centrosomes. However, it was not determined whether fusing GFP on the N-terminus of Tax altered or not its transcriptional activity. Indeed, a previous work demonstrated that GFP-Tax proteins are severely impaired for CREB/ATF pathway activation [92]. More recently, a study presented conflicting results, suggesting that CREB rather than NF-κB activation is required for Tax-induced aneuploidy [93]. Which, (if any), of Tax-dependent transcriptional signaling pathway is needed for inducing aneuploidy is therefore still a matter of debate.

**Figure 5:**
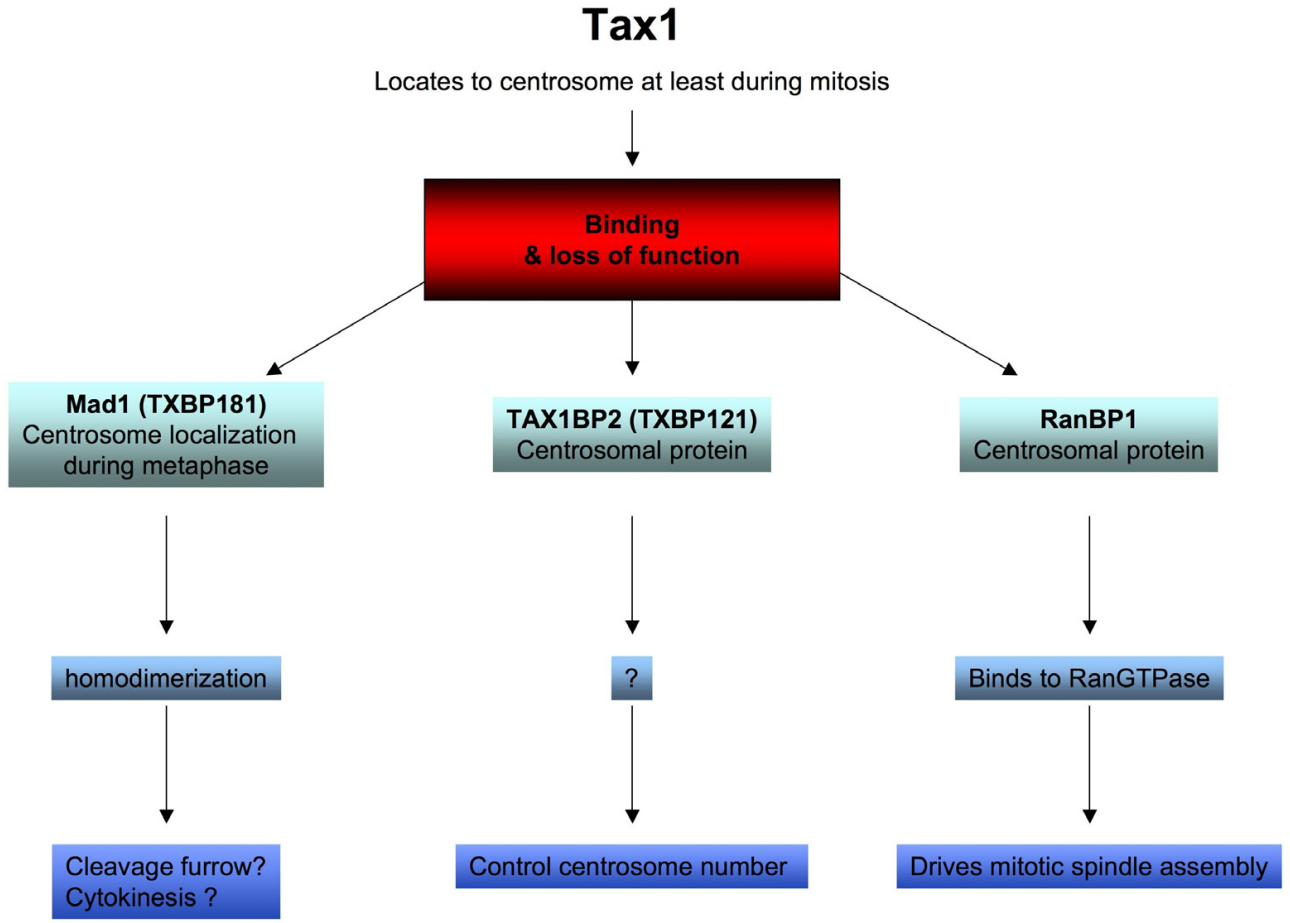
**Pleiotropic actions of Tax-1**. Tax-1 interacts and impairs the function of at least with 3 different centrosomal proteins (Mad1, TAX1BP2 and RanBP1) which participate in the control of mitosis.

Along with the results described above, Peloponese and coll. also proposed that the ability of Tax to bind RanBP1 (one of the major cytoplasmic effector of Ran) is a critical event for targeting the viral protein to the centrosome (Figure 5). However, this interaction is necessary but not sufficient for inducing aneuploidy [87]. Because it was previously shown that (i) RanBP1 is overexpressed in a number of transformed cell lines [94], and that (ii) ectopic expression of RanBP1 yields abnormal mitoses [95], it would be of interest to determine the level of expression of this protein in HTLV-1 transformed/immortalized cells vs. normal lymphocytes, but also whether Tax could transactivate the RanBP1 promoter. If this was the case, would NF-κB, as suggested by Peloponese, or CREB, as suggested by de la Fuente, be evoked?

The altered functions of several centrosomal proteins seems also to be involved in the Tax-driven aneuploidy. As an example, HsMAD1 (also known as TXBP181) functions are impaired in Tax expressing cells [85] (Figure 5). HsMAD1 acts at the G2-M-checkpoint. Since HsMAD1 localizes to the centrosome during metaphase, it is tempting to speculate that the loss of HsMAD1 functions could be linked to the loss or to the modification of the centrosomal activity.

Lately, another partner of Tax, the centrosomal TAX1BP2 protein (also known as TXBP121) [96], was also implicated in the Tax-dependent initiation of aneuploidy [84] (Figure 5). Remarkably, the authors observed first at least a 5-fold difference in the number of Tax expressing JPX-9 cells that display centrosome-amplification vs. untreated JPX-9. This is significantly higher than the 2 fold increase observed by Nitta et al in the same experimental settings. Then, they demonstrated that Tax binds to and colocalizes with endogenous TAX1BP2, forming perinuclear dots. In the absence of Tax, the overexpression of TAX1BP2 leads to a reduction in the number of cells that contain supernumerary centrosomes. On the contrary, depletion of endogenous TAX1BP2 induces centrosome amplification. Therefore, Tax and TAX1BP2 have opposite effects. Besides, a Tax mutant that does not interact with TAX1BP2 can no longer induce centrosome duplication. The authors concluded that Tax targets TAX1BP2 to cause aneuploidy.

In the end, a series of questions remain to be solved: Do Tax, RanBP1 and TAX1BP2 form a complex? Alternatively, do Tax and TAX1BP2 compete for RanBP1-binding? How does Tax target TAX1BP2? What is the level of TAX1BP2 in HTLV-1 infected cells?

One important fact does not fit perfectly well with all these experimental results: ATLL development is not a rapid process but takes decades [97,98]. We therefore believe there is an alternative and more provocative interpretation for these results. It is possible that aneuploidy occurs normally in a stochastic manner in cells which will then die. When Tax is expressed, and because of its pleiotropic effects, these aneuploid cells would then undergo a series of processes, such as p53 transcriptional inhibition for example, that would circumvent apoptosis induction. In other words, Tax would not induce aneuploidy but rather would allow aneuploid cells to survive.

## III. Conclusion

Viruses have evolved different strategies to traffic within an infected cell. Active transport along the cytoskeleton networks, in particular the MTs, has been demonstrated for a series of nuclear replicating viruses such as retroviruses. For these latter, the centrosome seems to play a central role both during early and late stages of the replication cycle. It will be important to understand the functional meaning of the centrosomal localization of incoming FV and HIV-1 following infection. Is it just a mandatory/compulsory route to reach the nucleus following trafficking along the MT network, or is it (also) a transforming platform which selectively modifies the incoming viral material thus allowing a successful integration into the host genome ?

In addition, the centrosome is not a mere spectator of the cell cycle but exerts a significant control over it. By providing a scaffold for many cell cycle regulators and their activity, it influences cell-cycle progression, especially during the G1 to S-phase transition [99,100]. To this end, this organelle receives and integrates signals from outside the cell and facilitates their conversion into cellular functions.

By targeting the centrosome, some viruses hijack its functions, leading eventually either to cell death or to cell transformation [101-106].

The role of this central organelle in retrovirus replication and pathogenicity is still mysterious and will certainly require more consideration.

## Abbreviations

HTLV-1: Human T cell leukemia virus type 1

HIV-1: Human Immunodeficiency virus type 1

ChK: C-terminal Src kinase-homologous kinase

MTOC: Microtubule organizing center

Cdk: Cyclin-dependent kinase

ATLL: Adult T cell leukemia/lymphoma

SRF: Serum responsive factor

CREB: cAMP response element-binding protein

MPF: Mitosis promoting factor

Vpr: Viral protein R

MAD1: Mitotic arrest-defective 1

Cdc: Cell division cycle

MEF: mouse embryonic fibroblasts

PCM: pericentriolar material

MT: microtubule

FV: Foamy Virus

hnRNP: A2: heterogeneous nuclear ribonucleoprotein A2

CTRS: cytoplasmic targeting-retention signal

M-PMV: Mason-Pfizer Monkey Virus

JSRV: Jaagsiekte Sheep Retrovirus

## Competing interests

The author(s) declare that they have no competing interests.

## Authors' contributions

PA, AZ, AS and RM wrote the manuscript. All authors have read and approved the manuscript.

## References

[B1] Boveri T (1902). Über merhrpolige Mitosen als Mittel zur analyse des zellkerns.. Verh D Phys Med Ges Würzburg NF.

[B2] Andersen JS, Wilkinson CJ, Mayor T, Mortensen P, Nigg EA, Mann M (2003). Proteomic characterization of the human centrosome by protein correlation profiling. Nature.

[B3] Fukasawa K (2005). Centrosome amplification, chromosome instability and cancer development. Cancer Lett.

[B4] Burkhard P, Stetefeld J, Strelkov SV (2001). Coiled coils: a highly versatile protein folding motif. Trends Cell Biol.

[B5] Tsou MF, Stearns T (2006). Controlling centrosome number: licenses and blocks. Curr Opin Cell Biol.

[B6] Tsou MF, Stearns T (2006). Mechanism limiting centrosome duplication to once per cell cycle. Nature.

[B7] Kuriyama R, Borisy GG (1981). Microtubule-nucleating activity of centrosomes in Chinese hamster ovary cells is independent of the centriole cycle but coupled to the mitotic cycle. J Cell Biol.

[B8] Hinchcliffe EH, Li C, Thompson EA, Maller JL, Sluder G (1999). Requirement of Cdk2-cyclin E activity for repeated centrosome reproduction in Xenopus egg extracts. Science.

[B9] Lacey KR, Jackson PK, Stearns T (1999). Cyclin-dependent kinase control of centrosome duplication. Proc Natl Acad Sci U S A.

[B10] Blow JJ, Dutta A (2005). Preventing re-replication of chromosomal DNA. Nat Rev Mol Cell Biol.

[B11] Nigg EA (2001). Mitotic kinases as regulators of cell division and its checkpoints. Nat Rev Mol Cell Biol.

[B12] Brinkley BR (2001). Managing the centrosome numbers game: from chaos to stability in cancer cell division. Trends Cell Biol.

[B13] Doxsey S, McCollum D, Theurkauf W (2005). Centrosomes in cellular regulation. Annu Rev Cell Dev Biol.

[B14] Doxsey S, Zimmerman W, Mikule K (2005). Centrosome control of the cell cycle. Trends Cell Biol.

[B15] Picard A, Karsenti E, Dabauvalle MC, Doree M (1987). Release of mature starfish oocytes from interphase arrest by microinjection of human centrosomes. Nature.

[B16] Perez-Mongiovi D, Beckhelling C, Chang P, Ford CC, Houliston E (2000). Nuclei and microtubule asters stimulate maturation/M phase promoting factor (MPF) activation in Xenopus eggs and egg cytoplasmic extracts. J Cell Biol.

[B17] Jackman M, Lindon C, Nigg EA, Pines J (2003). Active cyclin B1-Cdk1 first appears on centrosomes in prophase. Nat Cell Biol.

[B18] Han SJ, Conti M (2006). New pathways from PKA to the Cdc2/cyclin B complex in oocytes: Wee1B as a potential PKA substrate. Cell Cycle.

[B19] Raleigh JM, O'Connell MJ (2000). The G(2) DNA damage checkpoint targets both Wee1 and Cdc25. J Cell Sci.

[B20] Ghadimi BM, Sackett DL, Difilippantonio MJ, Schrock E, Neumann T, Jauho A, Auer G, Ried T (2000). Centrosome amplification and instability occurs exclusively in aneuploid, but not in diploid colorectal cancer cell lines, and correlates with numerical chromosomal aberrations. Genes Chromosomes Cancer.

[B21] Lingle WL, Lutz WH, Ingle JN, Maihle NJ, Salisbury JL (1998). Centrosome hypertrophy in human breast tumors: implications for genomic stability and cell polarity. Proc Natl Acad Sci U S A.

[B22] Pihan GA, Purohit A, Wallace J, Knecht H, Woda B, Quesenberry P, Doxsey SJ (1998). Centrosome defects and genetic instability in malignant tumors. Cancer Res.

[B23] Kops GJ, Weaver BA, Cleveland DW (2005). On the road to cancer: aneuploidy and the mitotic checkpoint. Nat Rev Cancer.

[B24] Hahn WC, Counter CM, Lundberg AS, Beijersbergen RL, Brooks MW, Weinberg RA (1999). Creation of human tumour cells with defined genetic elements. Nature.

[B25] Li R, Yerganian G, Duesberg P, Kraemer A, Willer A, Rausch C, Hehlmann R (1997). Aneuploidy correlated 100% with chemical transformation of Chinese hamster cells. Proc Natl Acad Sci U S A.

[B26] Marx J (2002). Debate surges over the origins of genomic defects in cancer. Science.

[B27] Doak SH, Jenkins GJ, Parry EM, Griffiths AP, Baxter JN, Parry JM (2004). Differential expression of the MAD2, BUB1 and HSP27 genes in Barrett's oesophagus-their association with aneuploidy and neoplastic progression. Mutat Res.

[B28] Duensing S, Munger K (2004). Mechanisms of genomic instability in human cancer: insights from studies with human papillomavirus oncoproteins. Int J Cancer.

[B29] Ried T, Heselmeyer-Haddad K, Blegen H, Schrock E, Auer G (1999). Genomic changes defining the genesis, progression, and malignancy potential in solid human tumors: a phenotype/genotype correlation. Genes Chromosomes Cancer.

[B30] Weaver BA, Cleveland DW (2006). Does aneuploidy cause cancer?. Curr Opin Cell Biol.

[B31] Rasnick D (2002). Aneuploidy theory explains tumor formation, the absence of immune surveillance, and the failure of chemotherapy. Cancer Genet Cytogenet.

[B32] Weaver BA, Silk AD, Montagna C, Verdier-Pinard P, Cleveland DW (2007). Aneuploidy acts both oncogenically and as a tumor suppressor. Cancer Cell.

[B33] Suzuki Y, Craigie R (2007). The road to chromatin - nuclear entry of retroviruses. Nat Rev Microbiol.

[B34] Greber UF, Way M (2006). A superhighway to virus infection. Cell.

[B35] Radtke K, Dohner K, Sodeik B (2006). Viral interactions with the cytoskeleton: a hitchhiker's guide to the cell. Cell Microbiol.

[B36] Dohner K, Sodeik B (2005). The role of the cytoskeleton during viral infection. Curr Top Microbiol Immunol.

[B37] Petit C, Giron ML, Tobaly-Tapiero J, Bittoun P, Real E, Jacob Y, Tordo N, De The H, Saib A (2003). Targeting of incoming retroviral Gag to the centrosome involves a direct interaction with the dynein light chain 8. J Cell Sci.

[B38] Saib A, Puvion-Dutilleul F, Schmid M, Peries J, de The H (1997). Nuclear targeting of incoming human foamy virus Gag proteins involves a centriolar step. J Virol.

[B39] Fackler OT, Krausslich HG (2006). Interactions of human retroviruses with the host cell cytoskeleton. Curr Opin Microbiol.

[B40] McDonald D, Vodicka MA, Lucero G, Svitkina TM, Borisy GG, Emerman M, Hope TJ (2002). Visualization of the intracellular behavior of HIV in living cells. J Cell Biol.

[B41] Arhel N, Genovesio A, Kim KA, Miko S, Perret E, Olivo-Marin JC, Shorte S, Charneau P (2006). Quantitative four-dimensional tracking of cytoplasmic and nuclear HIV-1 complexes. Nat Methods.

[B42] Igakura T, Stinchcombe JC, Goon PK, Taylor GP, Weber JN, Griffiths GM, Tanaka Y, Osame M, Bangham CR (2003). Spread of HTLV-I between lymphocytes by virus-induced polarization of the cytoskeleton. Science.

[B43] Yu SF, Eastman SW, Linial ML (2006). Foamy virus capsid assembly occurs at a pericentriolar region through a cytoplasmic targeting/retention signal in Gag. Traffic.

[B44] Choi G, Park S, Choi B, Hong S, Lee J, Hunter E, Rhee SS (1999). Identification of a cytoplasmic targeting/retention signal in a retroviral Gag polyprotein. J Virol.

[B45] Rhee SS, Hunter E (1987). Myristylation is required for intracellular transport but not for assembly of D-type retrovirus capsids. J Virol.

[B46] Sfakianos JN, LaCasse RA, Hunter E (2003). The M-PMV cytoplasmic targeting-retention signal directs nascent Gag polypeptides to a pericentriolar region of the cell. Traffic.

[B47] Sfakianos JN, Hunter E (2003). M-PMV capsid transport is mediated by Env/Gag interactions at the pericentriolar recycling endosome. Traffic.

[B48] Rhee SS, Hunter E (1990). A single amino acid substitution within the matrix protein of a type D retrovirus converts its morphogenesis to that of a type C retrovirus. Cell.

[B49] Murcia PR, Arnaud F, Palmarini M (2007). The Transdominant Endogenous Retrovirus enJS56A1 Associates with and Blocks Intracellular Trafficking of the JSRV Gag. J Virol.

[B50] Levesque K, Halvorsen M, Abrahamyan L, Chatel-Chaix L, Poupon V, Gordon H, DesGroseillers L, Gatignol A, Mouland AJ (2006). Trafficking of HIV-1 RNA is mediated by heterogeneous nuclear ribonucleoprotein A2 expression and impacts on viral assembly. Traffic.

[B51] Poole E, Strappe P, Mok HP, Hicks R, Lever AM (2005). HIV-1 Gag-RNA interaction occurs at a perinuclear/centrosomal site; analysis by confocal microscopy and FRET. Traffic.

[B52] Brown CR, Doxsey SJ, Hong-Brown LQ, Martin RL, Welch WJ (1996). Molecular chaperones and the centrosome. A role for TCP-1 in microtubule nucleation. J Biol Chem.

[B53] Brown CR, Hong-Brown LQ, Doxsey SJ, Welch WJ (1996). Molecular chaperones and the centrosome. A role for HSP 73 in centrosomal repair following heat shock treatment. J Biol Chem.

[B54] Hong S, Choi G, Park S, Chung AS, Hunter E, Rhee SS (2001). Type D retrovirus Gag polyprotein interacts with the cytosolic chaperonin TRiC. J Virol.

[B55] Minemoto Y, Shimura M, Ishizaka Y, Masamune Y, Yamashita K (1999). Multiple centrosome formation induced by the expression of Vpr gene of human immunodeficiency virus. Biochem Biophys Res Commun.

[B56] Watanabe N, Yamaguchi T, Akimoto Y, Rattner JB, Hirano H, Nakauchi H (2000). Induction of M-phase arrest and apoptosis after HIV-1 Vpr expression through uncoupling of nuclear and centrosomal cycle in HeLa cells. Exp Cell Res.

[B57] Chang F, Re F, Sebastian S, Sazer S, Luban J (2004). HIV-1 Vpr induces defects in mitosis, cytokinesis, nuclear structure, and centrosomes. Mol Biol Cell.

[B58] Glover DM, Hagan IM, Tavares AA (1998). Polo-like kinases: a team that plays throughout mitosis. Genes Dev.

[B59] Ohkura H, Hagan IM, Glover DM (1995). The conserved Schizosaccharomyces pombe kinase plo1, required to form a bipolar spindle, the actin ring, and septum, can drive septum formation in G1 and G2 cells. Genes Dev.

[B60] Jowett JB, Planelles V, Poon B, Shah NP, Chen ML, Chen IS (1995). The human immunodeficiency virus type 1 vpr gene arrests infected T cells in the G2 + M phase of the cell cycle. J Virol.

[B61] Sakai K, Dimas J, Lenardo MJ (2006). The Vif and Vpr accessory proteins independently cause HIV-1-induced T cell cytopathicity and cell cycle arrest. Proc Natl Acad Sci U S A.

[B62] Poon B, Grovit-Ferbas K, Stewart SA, Chen IS (1998). Cell cycle arrest by Vpr in HIV-1 virions and insensitivity to antiretroviral agents. Science.

[B63] Di Marzio P, Choe S, Ebright M, Knoblauch R, Landau NR (1995). Mutational analysis of cell cycle arrest, nuclear localization and virion packaging of human immunodeficiency virus type 1 Vpr. J Virol.

[B64] Zhou Y, Lu Y, Ratner L (1998). Arginine residues in the C-terminus of HIV-1 Vpr are important for nuclear localization and cell cycle arrest. Virology.

[B65] Zhou Y, Ratner L (2000). Phosphorylation of human immunodeficiency virus type 1 Vpr regulates cell cycle arrest. J Virol.

[B66] He J, Choe S, Walker R, Di Marzio P, Morgan DO, Landau NR (1995). Human immunodeficiency virus type 1 viral protein R (Vpr) arrests cells in the G2 phase of the cell cycle by inhibiting p34cdc2 activity. J Virol.

[B67] Re F, Braaten D, Franke EK, Luban J (1995). Human immunodeficiency virus type 1 Vpr arrests the cell cycle in G2 by inhibiting the activation of p34cdc2-cyclin B. J Virol.

[B68] Elder RT, Benko Z, Zhao Y (2002). HIV-1 VPR modulates cell cycle G2/M transition through an alternative cellular mechanism other than the classic mitotic checkpoints. Front Biosci.

[B69] Goh WC, Manel N, Emerman M (2004). The human immunodeficiency virus Vpr protein binds Cdc25C: implications for G2 arrest. Virology.

[B70] Elder RT, Yu M, Chen M, Zhu X, Yanagida M, Zhao Y (2001). HIV-1 Vpr induces cell cycle G2 arrest in fission yeast (Schizosaccharomyces pombe) through a pathway involving regulatory and catalytic subunits of PP2A and acting on both Wee1 and Cdc25. Virology.

[B71] Hrimech M, Yao XJ, Branton PE, Cohen EA (2002). Human immunodeficiency virus type 1 Vpr-mediated G(2) cell cycle arrest: Vpr interferes with cell cycle signaling cascades by interacting with the B subunit of serine/threonine protein phosphatase 2A. Embo J.

[B72] Peng CY, Graves PR, Thoma RS, Wu Z, Shaw AS, Piwnica-Worms H (1997). Mitotic and G2 checkpoint control: regulation of 14-3-3 protein binding by phosphorylation of Cdc25C on serine-216. Science.

[B73] Forrest A, Gabrielli B (2001). Cdc25B activity is regulated by 14-3-3. Oncogene.

[B74] Kino T, Gragerov A, Valentin A, Tsopanomihalou M, Ilyina-Gragerova G, Erwin-Cohen R, Chrousos GP, Pavlakis GN (2005). Vpr protein of human immunodeficiency virus type 1 binds to 14-3-3 proteins and facilitates complex formation with Cdc25C: implications for cell cycle arrest. J Virol.

[B75] Muthumani K, Choo AY, Premkumar A, Hwang DS, Thieu KP, Desai BM, Weiner DB (2005). Human immunodeficiency virus type 1 (HIV-1) Vpr-regulated cell death: insights into mechanism. Cell Death Differ.

[B76] Grassmann R, Aboud M, Jeang KT (2005). Molecular mechanisms of cellular transformation by HTLV-1 Tax. Oncogene.

[B77] Schmitt I, Rosin O, Rohwer P, Gossen M, Grassmann R (1998). Stimulation of cyclin-dependent kinase activity and G1- to S-phase transition in human lymphocytes by the human T-cell leukemia/lymphotropic virus type 1 Tax protein. J Virol.

[B78] Haller K, Wu Y, Derow E, Schmitt I, Jeang KT, Grassmann R (2002). Physical interaction of human T-cell leukemia virus type 1 Tax with cyclin-dependent kinase 4 stimulates the phosphorylation of retinoblastoma protein. Mol Cell Biol.

[B79] Neuveut C, Low KG, Maldarelli F, Schmitt I, Majone F, Grassmann R, Jeang KT (1998). Human T-cell leukemia virus type 1 Tax and cell cycle progression: role of cyclin D-cdk and p110Rb. Mol Cell Biol.

[B80] Kehn K, Fuente Cde L, Strouss K, Berro R, Jiang H, Brady J, Mahieux R, Pumfery A, Bottazzi ME, Kashanchi F (2005). The HTLV-I Tax oncoprotein targets the retinoblastoma protein for proteasomal degradation. Oncogene.

[B81] Park HU, Jeong JH, Chung JH, Brady JN (2004). Human T-cell leukemia virus type 1 Tax interacts with Chk1 and attenuates DNA-damage induced G2 arrest mediated by Chk1. Oncogene.

[B82] Park HU, Jeong SJ, Jeong JH, Chung JH, Brady JN (2006). Human T-cell leukemia virus type 1 Tax attenuates gamma-irradiation-induced apoptosis through physical interaction with Chk2. Oncogene.

[B83] Liu B, Hong S, Tang Z, Yu H, Giam CZ (2005). HTLV-I Tax directly binds the Cdc20-associated anaphase-promoting complex and activates it ahead of schedule. Proc Natl Acad Sci U S A.

[B84] Ching YP, Chan SF, Jeang KT, Jin DY (2006). The retroviral oncoprotein Tax targets the coiled-coil centrosomal protein TAX1BP2 to induce centrosome overduplication. Nat Cell Biol.

[B85] Jin DY, Spencer F, Jeang KT (1998). Human T cell leukemia virus type 1 oncoprotein Tax targets the human mitotic checkpoint protein MAD1. Cell.

[B86] Nitta T, Kanai M, Sugihara E, Tanaka M, Sun B, Nagasawa T, Sonoda S, Saya H, Miwa M (2006). Centrosome amplification in adult T-cell leukemia and human T-cell leukemia virus type 1 Tax-induced human T cells. Cancer Sci.

[B87] Peloponese JM, Haller K, Miyazato A, Jeang KT (2005). Abnormal centrosome amplification in cells through the targeting of Ran-binding protein-1 by the human T cell leukemia virus type-1 Tax oncoprotein. Proc Natl Acad Sci U S A.

[B88] Pumfery A, de la Fuente C, Kashanchi F (2006). HTLV-1 Tax: centrosome amplification and cancer. Retrovirology.

[B89] Itoyama T, Chaganti RS, Yamada Y, Tsukasaki K, Atogami S, Nakamura H, Tomonaga M, Ohshima K, Kikuchi M, Sadamori N (2001). Cytogenetic analysis and clinical significance in adult T-cell leukemia/lymphoma: a study of 50 cases from the human T-cell leukemia virus type-1 endemic area, Nagasaki. Blood.

[B90] Shimoyama M, Abe T, Miyamoto K, Minato K, Tobinai K, Nagoshi H, Matsunaga M, Nomura T, Tsubota T, Ohnoshi T (1987). Chromosome aberrations and clinical features of adult T cell leukemia-lymphoma not associated with human T cell leukemia virus type I. Blood.

[B91] Nagata K, Ohtani K, Nakamura M, Sugamura K (1989). Activation of endogenous c-fos proto-oncogene expression by human T-cell leukemia virus type I-encoded p40tax protein in the human T-cell line, Jurkat. J Virol.

[B92] Meertens L, Pise-Masison C, Quere N, Brady J, Gessain A, Mahieux R (2004). Utilization of the CBP but not the p300 co-activator by human T-lymphotropic virus type-2 Tax for p53 inhibition. Oncogene.

[B93] de la Fuente C, Gupta MV, Klase Z, Strouss K, Cahan P, McCaffery T, Galante A, Soteropoulos P, Pumfery A, Fujii M, Kashanchi F (2006). Involvement of HTLV-I Tax and CREB in aneuploidy: a bioinformatics approach. Retrovirology.

[B94] Di Matteo G, Fuschi P, Zerfass K, Moretti S, Ricordy R, Cenciarelli C, Tripodi M, Jansen-Durr P, Lavia P (1995). Transcriptional control of the Htf9-A/RanBP-1 gene during the cell cycle. Cell Growth Differ.

[B95] Guarguaglini G, Renzi L, D'Ottavio F, Di Fiore B, Casenghi M, Cundari E, Lavia P (2000). Regulated Ran-binding protein 1 activity is required for organization and function of the mitotic spindle in mammalian cells in vivo. Cell Growth Differ.

[B96] Chun AC, Zhou Y, Wong CM, Kung HF, Jeang KT, Jin DY (2000). Coiled-coil motif as a structural basis for the interaction of HTLV type 1 Tax with cellular cofactors. AIDS Res Hum Retroviruses.

[B97] Matsuoka M (2003). Human T-cell leukemia virus type I and adult T-cell leukemia. Oncogene.

[B98] Matsuoka M, Jeang KT (2007). Human T-cell leukaemia virus type 1 (HTLV-1) infectivity and cellular transformation. Nat Rev Cancer.

[B99] Hinchcliffe EH, Miller FJ, Cham M, Khodjakov A, Sluder G (2001). Requirement of a centrosomal activity for cell cycle progression through G1 into S phase. Science.

[B100] Khodjakov A, Rieder CL (2001). Centrosomes enhance the fidelity of cytokinesis in vertebrates and are required for cell cycle progression. J Cell Biol.

[B101] Duensing S, Munger K (2002). Human papillomaviruses and centrosome duplication errors: modeling the origins of genomic instability. Oncogene.

[B102] Forgues M, Difilippantonio MJ, Linke SP, Ried T, Nagashima K, Feden J, Valerie K, Fukasawa K, Wang XW (2003). Involvement of Crm1 in hepatitis B virus X protein-induced aberrant centriole replication and abnormal mitotic spindles. Mol Cell Biol.

[B103] Jouvenet N, Wileman T (2005). African swine fever virus infection disrupts centrosome assembly and function. J Gen Virol.

[B104] Miki R, Okuda M, Oikawa T, Watanabe M, Ma Z, Matsumoto K, Iwata H, Inokuma H (2004). Centrosome amplification and chromosomal instability in feline lymphoma cell lines. J Vet Med Sci.

[B105] Pan H, Zhou F, Gao SJ (2004). Kaposi's sarcoma-associated herpesvirus induction of chromosome instability in primary human endothelial cells. Cancer Res.

[B106] Zhao LY, Liao D (2003). Sequestration of p53 in the cytoplasm by adenovirus type 12 E1B 55-kilodalton oncoprotein is required for inhibition of p53-mediated apoptosis. J Virol.

[B107] Duensing S, Munger K (2001). Centrosome abnormalities, genomic instability and carcinogenic progression. Biochim Biophys Acta.

